# Investigation of Inter-Slice Magnetization Transfer Effects as a New Method for MTR Imaging of the Human Brain

**DOI:** 10.1371/journal.pone.0117101

**Published:** 2015-02-09

**Authors:** Jeffrey W. Barker, Paul Kyu Han, Seung Hong Choi, Kyongtae Ty Bae, Sung-Hong Park

**Affiliations:** 1 Department of Radiology, University of Pittsburgh, Pittsburgh, Pennsylvania, United States of America; 2 Department of Bioengineering, University of Pittsburgh, Pittsburgh, Pennsylvania, United States of America; 3 Department of Bio and Brain Engineering, Korea Advanced Institute of Science and Technology, Daejeon, South Korea; 4 Department of Radiology, Seoul National University College of Medicine, Seoul, South Korea; West China Hospital of Sichuan University, CHINA

## Abstract

We present a new method for magnetization transfer (MT) ratio imaging in the brain that requires no separate saturation pulse. Interslice MT effects that are inherent to multi-slice balanced steady-state free precession (bSSFP) imaging were controlled via an interslice delay time to generate MT-weighted (0 s delay) and reference images (5–8 s delay) for MT ratio (MTR) imaging of the brain. The effects of varying flip angle and phase encoding (PE) order were investigated experimentally in normal, healthy subjects. Values of up to ∼50% and ∼40% were observed for white and gray matter MTR. Centric PE showed larger MTR, higher SNR, and better contrast between white and gray matter than linear PE. Simulations of a two-pool model of MT agreed well with *in vivo* MTR values. Simulations were also used to investigate the effects of varying acquisition parameters, and the effects of varying flip angle, PE steps, and interslice delay are discussed. Lastly, we demonstrated reduced banding with a non-balanced SSFP-FID sequence and showed preliminary results of interslice MTR imaging of meningioma.

## Introduction

Protons that are bound to macromolecules can exchange magnetization with free water protons leading to magnetization transfer (MT) phenomena [1]. Macromolecular protons cannot be observed directly with magnetic resonance imaging (MRI) because of fast transverse relaxation (T_2_ ∼ 10 *μ*s); however, macromolecular protons can be preferentially saturated by off-resonance (with respect to free water) radio frequency (RF) irradiation, since the absorption spectrum of macromolecular protons is much broader than that of free water. Exchange between the two pools of protons can transfer an observable decrease in magnetization to the free water pool. The percent signal decrease due to MT is called the magnetization transfer ratio (MTR). Changes in MTR can often give information about the macromolecules involved in generating the MT effects. One of the major applications of MTR imaging has been the evaluation of white matter (WM) integrity in multiple sclerosis [2], in which myelin content has been found to be significantly correlated with MTR [3]. Other applications include imaging articular cartilage of the knee [4–6], intervertebral disc degeneration [7], and characterization of brain tumors [8, 9].

Interslice MT effects that are inherent to sequential multi-slice acquisitions [10–13] have been used to generate contrast for MT asymmetry imaging without a separate saturation pulse in the alternate ascending/descending directional navigation (ALADDIN) pulse sequence [14]. We hypothesize that this approach may be used for MTR imaging with an additional acquisition of reference images without MT weighting. The purpose of this work was to demonstrate the feasibility of MTR imaging in the brain using interslice MT effects to generate MT contrast and to investigate the characteristics of interslice MTR image acquisition with multi-slice balanced steady state free precession (bSSFP) imaging.

In this study, we validated the source of image contrast using a 10% agar phantom (high MT effects) and saline phantom (no MT effects) and by comparing *in vivo* MTR images of the brain acquired with the proposed interslice method to MTR images acquired with conventional presaturation pulses. We compared *in vivo* MTR values for gray matter (GM) and WM at varying flip angle and phase encoding (PE) order with predictions from numerical simulations of the two-pool model using tissue parameters from the literature. Simulations were also used to investigate the effects of varying the number of PE steps, flip angle, and interslice delay as well as the accumulation of MT effects over multiple slices. Lastly, we demonstrate reduced banding with non-balanced SSFP and applied the proposed interslice MTR imaging method to a meningioma patient.

## Materials and Methods

### Theory

**Fig 1:**
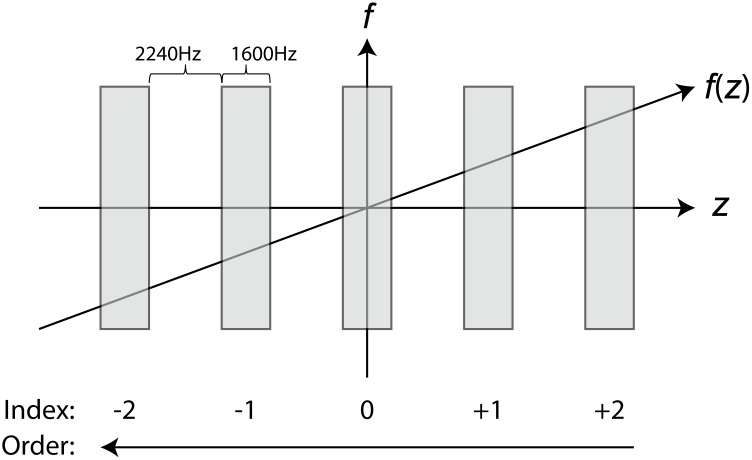
Illustration of interslice MT effects. The application of a gradient varies the Larmor frequency *f*(*z*) linearly in space (*z*). During excitation, the slice of interest (slice 0) receives on resonance excitation. With a positive gradient polarity and descending slice order (shown above), the next slice to be acquired (slice −1) receives off-resonance irradiation at a frequency offsets of 3840 Hz.

In 2D sequences, slice-selection is achieved by applying a linear gradient perpendicular to the slice plane causing the Larmor precession frequency to vary as a function of position. Excitation of a slice of interest is achieved by adjusting RF-pulse frequency to the Larmor frequency of the desired plane. The slice of interest receives on-resonance excitation; however, the rest of the volume receives off-resonance irradiation. During acquisition of one slice, excitation pulses are effectively a series of off-resonance saturation pulses to future slices (Fig. 1). The off-resonance frequency received by neighboring slices is given by

$$\delta_{n}=-BW\cdot \left(1+\frac{GAP}{THK}\right)\cdot n\cdot sign(GRAD)\cdot ORD\;,$$ (1)

where *BW* is the bandwidth of the RF-pulse, *GAP* is the interslice gap; *THK* is the slice thickness; *n* is the slice index with positive indices indicating slices superior to the acquisition slice, and negative indices indicating slices inferior to the acquisition slice; *sign*(*GRAD*) is the sign of the gradient; *ORD* is +1 if ascending slice order and −1 if descending slice order. The off-resonance irradiation received by neighboring slices can saturate macromolecular protons leading to interslice MT effects. This idea is illustrated in Fig. 1.

Interslice MT effects can be enhanced as a mechanism for generating contrast by the use of bSSFP acquisition, in which high flip angle and short repetition time (TR) lead to high saturation of the macromolecular pool. The interslice gap is set to a high value (e.g. 140% the slice thickness) to avoid crosstalk caused by overlapping slice profiles and so that interleaving two acquisitions gives a full set of images at typical gap size (e.g. 20% the slice thickness) [14]. For imaging in the brain, descending slice order is preferred to ascending slice order, in order to suppress signal contributions from blood perfusion. Because MT effects can accumulate over multiple slices, a few extra “dummy” slices must be collected in the MT-weighted image sets to ensure homogeneous MT contrast across slices. These slices can be positioned outside the imaging volume of interest (e.g., above the head) and discarded during reconstruction. Reference images without MT-weighting can be acquired by adding an interslice delay sufficient for T_1_ recovery. The MTR value, which measures the percent signal decrease, can be calculated pixel by pixel as follows:

$$MTR=(I_{Ref}-I_{MT})/I_{Ref}\;\times 100\%\;,$$ (2)

where *I*_*MT*_ and *I*_*Ref*_ are the signal intensities of corresponding pixels in the MT-weighted and reference images, respectively.

The modified two-pool model [15] can be used to quantitatively model MT effects generated by sequential 2D bSSFP acquisitions. In this model, a free liquid pool (subscript *f*) exchanges longitudinal magnetization with a restricted macromolecular pool (subscript *r*). Using an RF-pulse given by

$$B_{1}(t)=b_{1}(t)\cos\left((\omega_{RF}-\omega_{0,f})\;t\right)\hat{x}+b_{1}(t)\sin\left((\omega_{RF}-\omega_{0,f})\;t\right)\hat{y}\;,$$ (3)

where *ω*_*RF*_ is the angular frequency of the RF pulse, *ω*_0, *f*_ is the angular frequency at free water resonace, and *b*_1_(*t*) is the magnitude of the RF pulse, the two pool model can be described mathematically in a frame rotating at *ω*_0, *f*_ using the Bloch equations with additional terms for exchange of longitudinal magnetization between the proton pools:

$$\frac{dM_{x,f}}{dt}=\;\gamma b_{1}(t)\;M_{z,f}\;\sin\left((\omega_{RF}-\omega_{0,f})\;t\right)-\frac{M_{x,f}}{T_{2,f}}$$ (4)

$$\frac{dM_{y,f}}{dt}=\;\gamma b_{1}(t)\;M_{z,f}\;\cos\left((\omega_{RF}-\omega_{0,f})\;t\right)-\frac{M_{y,f}}{T_{2,f}}$$ (5)

$$\begin{array}{cc} \frac{dM_{z,f}}{dt}= & -\gamma b_{1}(t)\;M_{x,f}\;\sin((\omega_{RF}-\omega_{0,f})\;t)\\ & -\gamma b_{1}(t)\;M_{y,f}\;\cos\left((\omega_{RF}-\omega_{0,f})\;t\right)\\ & -\frac{M_{z,f}-M_{0,f}}{T_{1,f}}-M_{z,f}\;F\;k_{r}+M_{z,r}\;k_{r} \end{array}$$ (6)

$$\frac{dM_{z,r}}{dt}=\;-\frac{M_{z,r}-M_{0,r}}{T_{1,r}}+M_{z,f}\;F\;k_{r}-M_{z,r}\;k_{r}-M_{z,r}\;W(t)\;,$$ (7)

with *z* denoting the longitudinal component, and *x* and *y* denoting transverse components of magnetization. The longitudinal and transverse magnetization decay constants are given by *T*_1_ and *T*_2_, respectively. The ratio of the fully-relaxed longitudinal magnetizations gives the ratio of pool sizes (*F* = *M*_0, *r*_/*M*_0, *f*_), and *k*_*r*_ is the pseudo-first-order exchange rate constant. The saturation rate of the restricted pool is proportional to the square of the RF-pulse amplitude and to the absorption lineshape, *G*(*ω*):

$$W(t)=\pi \;\gamma^{2}\;b_{1}^{2}(t)\;G(\omega_{RF})\;.$$ (8)

A super-Lorentzian lineshape has been found to model tissues well [16] and is defined by the following integral:

$$G(\omega )=T_{2,r}\sqrt{\frac{2}{\pi }}\int_{0}^{1}\frac{1}{\left|3u^{2}-1\right|}\;\exp\left(-2{\left(\frac{(\omega -\omega_{0,r})\;T_{2,r}}{3u^{2}-1}\right)}^{2}\right)du\;,$$ (9)

where the term *ω*_0, *r*_ is the peak of the absorption spectrum of the macromolecular pool, which can account for MT asymmetry when *ω*_0, *r*_ ≠ *ω*_0, *f*_[17]. For this study, MT asymmetry was not considered (*ω*_0, *r*_ = *ω*_0, *f*_). On-resonance MT effects [18] were simulated for WM and GM by setting *G*(0) = 1.4 × 10^−5^ s^−1^ according to Gloor, Scheffler, and Bieri [19].

### Ethical considerations

All imaging experiments were approved by the Institutional Review Boards at the University of Pittsburgh and Seoul National University and written informed consent was obtained from all participants.

### Instrumentation and Software

The experiments were performed on Siemens 3T Trio systems (Siemens Medical Solutions, Erlangen, Germany), and a 12-element head matrix coil was used for reception with body coil transmission for all data acquisitions. All simulations and analyses were performed with Matlab (Mathworks, Natick, MA).

### Image Reconstruction

Raw data were reconstructed to images by the MR scanners. Dummy slices in the MT-weighted images were discarded prior to MTR calculation. The number of MTR slices was equal to the the number of reference image slices for all acquisitions described below. Calculation of MTR images consisted of creating either a whole head or brain mask and calculating MTR pixel by pixel inside the masked region according to Eq. 2. Brain masks were created from segmentations generated using SPM8 software (Wellcome Trust Centre for Neuroimaging, London, UK), whereas whole head masks were generated simply by thresholding based on intensity.

### Simulations

Computer simulations were performed using the parameters summarized in Table 1 for GM and WM. We considered six prior slices of off-resonance saturation for MT-weighting (e.g., 23040 Hz, 19200 Hz, 15360 Hz, 11520 Hz, 7680 Hz, 3840 Hz), unless otherwise indicated. For reference image signal, the acquisition slice was simulated with no prior slices of off-resonance saturation (i.e., full T_1_ recovery from prior slices), except when specifically investigating the effects of varying interslice delay. We modeled excitation with a Gaussian windowed sinc pulse. Other simulation parameters were taken to match acquisition parameters, such as flip angle, number of PE steps, TR, and RF duration. The set of differential equations 4–7 were solved using the 4th/5th order Runge-Kutta algorithm. For analysis, we calculated MTR values using the magnitude of the transverse magnetization at the center line of k-space for MT-weighted and reference signal simulations. In addition to comparing simulations with *in vivo* data, we used simulations to investigate the dependence of MT contrast on the number of preceding slices for varying flip angles and number of PE steps. Lastly, we investigated the effects of varying the interslice delay from 0–8 s by simulating reference image acquisition with 6 prior slices of off-resonance irradiation with a specified delay time between each slice.

**Table 1:** Two-Pool MT Parameters at 3T.

	*T*_1, *f*_	*T*_2, *f*_	*T*_1, *f*_	*T*_2, *f*_	*k*_*r*_	*F*
White Matter	1.1 s	85 ms*	1 s	13 *μ*s	23 s^−1^	0.14
Gray Matter	1.8 s	99 ms	1 s	9 *μ*s	40 s^−1^	0.05

*Measured in this study. All other values from Stanisz et. al. [25].

### Phantom Imaging

A cylindrical 10% agar phantom with a diameter of 140 mm and height of 180 mm was imaged for initial assessment of interslice MTR imaging. Additionally, a cylindrical saline phantom with a diameter of 120 mm and height of 195 mm was imaged with the same acquisition parameters as a negative control. The MTR images were reconstructed from MT-weighted and reference bSSFP images according to Eq. 2. Centric PE order was used for acquisition. Other acquisition parameters were as follows: slice order = descending; slice-select gradient polarity = positive; readout gradient polarity = positive; TR/TE = 4.56/2.28 ms; matrix size = 256 × 256; field of view = 256 × 256 mm^2^; flip angle = 50°; slice thickness = 4 mm; interslice gap = 5.6 mm (0.8 mm after interleaving); scan direction = axial; PE direction = anterior-posterior; dummy PE steps = 30; phase oversampling = 50%; number of averages = 1; RF-pulse BW = 1600 Hz; acquisition BW = 501 Hz/pixel; number of slices = 19 and 18 for each interleaved MT-weighted image set (including 6 dummy slices each; 12 total) and 25 for reference images; interslice delay = 0 s for MT-weighted images and 6 s for reference images.

### Comparison of Interslice and Presaturation MT Effects

For three normal, healthy subjects (age 21–40), MTR images were acquired using the proposed interslice method and using conventional presaturation pulses with an identical bSSFP readout (single slice; no interslice MT effects), in order to confirm the source of image contrast *in vivo*. Three different off-resonance irradiation frequencies were used for the presaturation pulses corresponding to the offset frequencies of the first (3200 Hz), second (6400 Hz), and third prior (9600 Hz) slices of the interslice method. The average RF-power off-resonance irradiation of the two methods were equivalent (94 *μ*T). The following parameters of the bSSFP readout were the same for both acquistion methods: TR/TE = 4.15/2.08 ms; matrix size = 128 × 128; field of view = 220 × 220 mm^2^; flip angle = 60°; slice thickness = 5 mm; phase partial Fourier = 6/8; scan direction = axial; PE direction = right-left; initial dummy PE steps = 30; centric PE order; phase oversampling = 0%; RF-pulse BW = 1333 Hz; and acquisition BW = 592 Hz/pixel.

The interslice MTR images were acquired with descending slice order and positive slice-select gradient. The rest of the imaging parameters for the proposed interslice method were as follows: gap = 7 mm; number of average = 1; number of slices = 19 (including 6 dummy slices) for MT-weighted images and 13 for reference images; interslice delay = 0 s for MT-weighted images and 5 s for reference images; nominal scan time of 10 s for MT-weighted images and scan time of ∼1.1 min for reference images. The off-resonance irradiation condition of the interslice method was: pulse width = 1.2 ms; inter-pulse interval = 4.15 ms (∼29% duty cycle); and average RF power = 0.94 *μ*T.

For the images acquired with presaturation, both MT-weighted and reference images were acquired with number of slices = 1, number of averages = 1, and a sufficient acquisition delay time of 5 s to get rid of any residual signals prior to each measurement. A pulse train of 75 Gaussian pulses were used for off-resonance irradiation with the following saturation condition: flip angle = 578.4°; pulse width = 20 ms; inter-pulse interval = 40 ms (50% duty cycle); total saturation duration = 3 s; average RF power = 0.94 *μ*T (equivalent to interslice method above); off-resonance irradiation frequencies = +3200 Hz, +6400 Hz, and +9600 Hz.

Regions of interest (ROI) for the data were created manually for WM. Mean MTR values were computed for the WM ROI for each subject, and the results were averaged across subjects. Signal to noise ratio (SNR) was estimated from the difference image (*I*_*Ref*_−*I*_*MT*_) as the mean of the signal in the WM ROI divided by the standard deviation of a large region in the difference image containing only noise.

### Effects of Varying Flip Angle and Phase Encoding Order

For six normal, healthy subjects (age 24–39), MTR images were reconstructed from MT-weighted and reference bSSFP images acquired at varying flip angles from 15° to 90° in 15° intervals with descending slice order and positive slice-select gradient. Additional scan parameters were as follows: TR/TE = 4.11/2.06 ms; matrix size = 128 × 128; field of view = 230 × 230 mm^2^; slice thickness = 5 mm; interslice gap = 7 mm; scan direction = axial; PE direction = anterior-posterior; initial dummy PE steps = 10/30 for linear/centric PE order; phase oversampling = 50%; number of averages = 1; RF-pulse BW = 1067 Hz; acquisition BW = 673 Hz/pixel; number of slices = 15 for MT-weighted images (including 8 dummy slices) and 7 for reference images; interslice delay = 0 s for MT-weighted images and 8 s for reference images. Scan time for MT-weighted images was 13 s and scan time for reference images was ∼1 min. Lastly, we measured the observed T_2_ value of the center slice using a multi-contrast spin echo sequence with echo times varying from 30 ms to 300 ms in 30 ms intervals.

Regions of interests for the data were created by automatic segmentation of GM and WM via SPM8 software. Segmentation results were checked manually to ensure quality. Mean MTR values were computed for the WM and GM ROIs as a function of flip angle for each subject, and the results were averaged across subjects. Signal to noise ratio was estimated from the difference image (*I*_*Ref*_−*I*_*MT*_) as the mean of the signal in the combined WM and GM ROI divided by the standard deviation of a large region in the difference image containing only noise.

### Comparison of bSSFP and SSFP-FID

For five normal, healthy subjects (ages 24–49), MTR images were acquired for near full brain coverage using a bSSFP sequence. Two of the subjects were also imaged with a SSFP-FID sequence. Common acquisition parameters for both sequences were as follows: slice order = descending; slice-select gradient polarity = positive; readout gradient polarity = positive; matrix size = 256 × 256; field of view = 256 × 256 mm^2^; flip angle = 50°; slice thickness = 3 mm; interslice gap = 4.2 mm (0.6 mm after interleaving); scan direction = axial; PE direction = anterior-posterior; dummy PE steps = 30; phase oversampling = 50%; number of averages = 1; RF-pulse BW = 1600 Hz; acquisition BW = 501 Hz/pixel; number of slices = 19 and 18 for each interleaved MT-weighted image set (including 6 dummy slices each; 12 total) and 25 for reference images; interslice delay = 0 s for MT-weighted images and 6 s for reference images. For the bSSFP sequence, TR/TE = 4.56/2.28 ms and total scan time = 4.36 min (1.17 min for MT-weighted images and 3.18 min for reference images). For the SSFP-FID sequence, TR/TE = 4.31/2.21 ms and total scan time = 4.24 min (1.10 min for MT-weighted images and 3.14 min for reference images).

For each subject ROIs were created manually for WM. Mean MTR and SNR for the WM ROI were estimated for each subject, and the results were averaged across subjects. The MTR and SNR values were compared for the bSSFP and SSFP-FID sequences.

### Interslice MTR Imaging of Meningioma

For a meningioma patient, MTR images were acquired with full brain coverage using acquisition parameters: PE order = centric; slice order = descending; slice-select gradient polarity = positive; readout gradient polarity = positive; TR/TE = 4.15/2.08 ms; matrix size = 128 × 128; field of view = 220 × 220 mm^2^; flip angle = 60°; slice thickness = 5 mm; interslice gap = 7 mm; scan direction = axial; PE direction = right-left; initial dummy PE steps = 30; phase oversampling = 0%; phase partial Fourier = 6/8; number of averages = 1; RF-pulse BW = 1333 Hz; acquisition BW = 592 Hz/pixel; number of slices = 18 and 19 for each interleaved MT-weighted image set (including 9 dummy slices each; 18 total) and 19 for reference images; interslice delay = 0 s for MT-weighted images and 5 s for reference images. The total scan time was 2.1 min (0.4 min for MT-weighted images and 1.7 min for reference images).

For comparison with the proposed method, T_2_-weighted images were acquired using a Turbo Spin Echo (TSE) sequence with imaging parameters: number of slices = 25; TR = 6000 ms; TE = 93 ms; echo train length = 18; matrix size = 640 × 520; field of view = 220 × 178 mm^2^; number of acquisitions = 1; slice thickness = 5 mm; flip angle = 120°; and scan time = ∼1.2 min.

## Results

### Phantom Imaging

**Fig 2:**
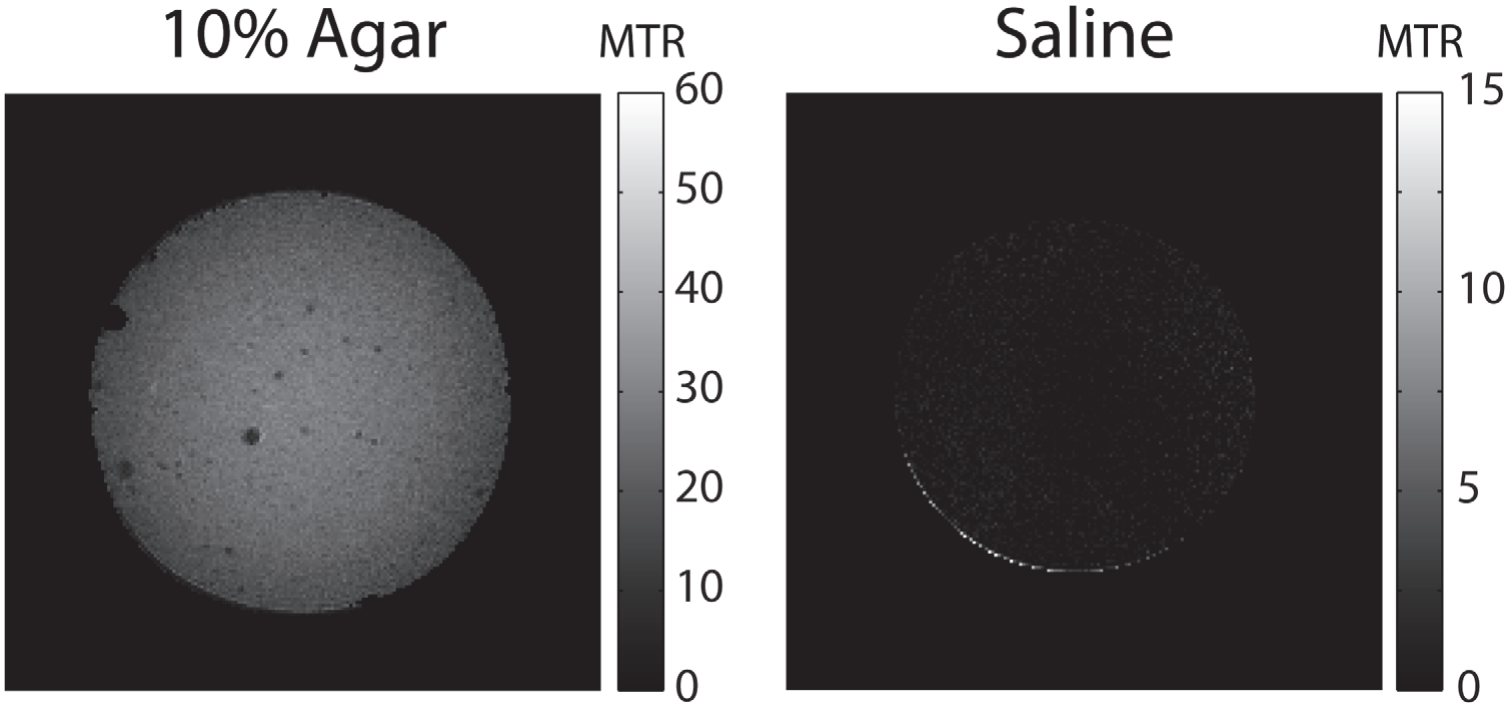
MTR images of a 10% agar phantom (left) and saline phantom (right).

Fig. 2 shows the resulting MTR images of the 10% agar phantom and saline phantom. The MTR images of the agar phantom (left) showed relatively homogeneous MTR values with the exception of dark spots caused by air bubbles trapped in the phantom. The saline phantom (right), which was expected to have no MT effects, was free from extraneous signals (note the scale difference between the two images). Together, the agar and control phantom images strongly support MT effects as the source of image contrast.

### Comparison of Interslice and Presaturation MT Effects

**Fig 3:**
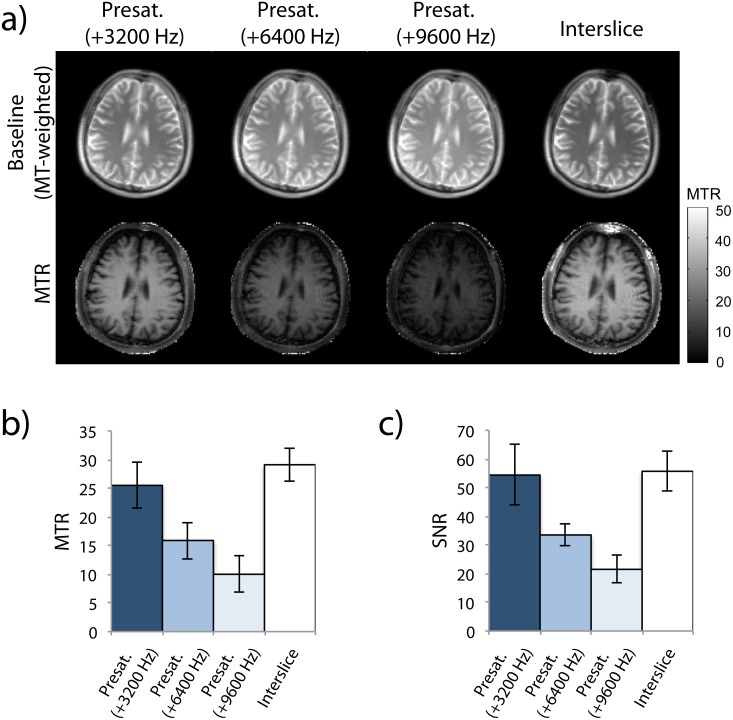
Comparison of MTR images generated with interslice MT effects and with presaturation. Offset irradiation frequencies of the presaturation pulses corresponded to the offeset frequencies of the first (+3200 Hz), second (+6400 Hz), and third (+9600 Hz) prior slices of the interslice method. Average RF-power of saturation was equivalent in both methods. Baseline (MT-weighted) and MTR images (a) from a representative subject are shown. Both MTR (b) and SNR (c) were calculated for white matter. Error bars show the 95% confidence interval of the group average.

Fig. 3 shows the representative MTR images using the interslice method and using conventional presaturation with a bSSFP readout for different offset irradiation frequencies corresponding to the offset frequencies of the first, second, and third prior slices of the interslice method. Visually, the signals in WM were higher than GM for all MTR images (Fig. 3a). The MTR images acquired with presaturation showed decreasing MTR and SNR for increasing offset irradiation frequencies. The average SNR and MTR values from the proposed interslice method were similar to those with presaturation at an offset irradiation frequency corresponding to the first prior slice of the interslice method (Fig. 3b–c), indicating that contribution of the first prior slice is dominant in the interslice MTR method and that the saturation efficiency of the interslice method is comparable to conventional method.

### Effects of Varying Flip Angle and Phase Encoding Order

**Fig 4:**
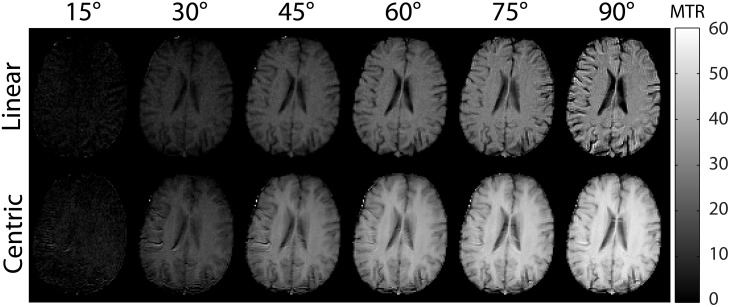
Center slices of MTR images from a representative subject are shown for varying flip angles and for linear phase encoding (top) and centric phase encoding (bottom).

Fig. 4 shows the center slice images for varying flip angle and for linear and centric PE from a representative subject. Centric PE images showed better GM and WM contrast, suggesting that relaxation effects influenced image contrast with linear PE.

**Fig 5:**
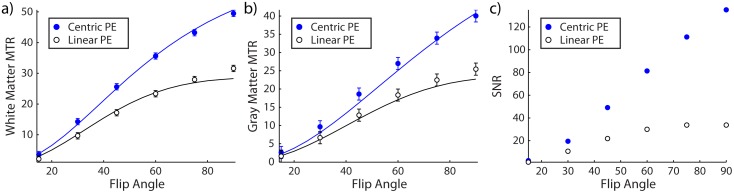
Mean MTR values across subjects from regions of interest for white (a) and gray matter (b). Predicted values from simulating the two-pool model (solid lines) with parameters from the literature show close agreement with the *in vivo* values. Centric phase encoding shows substantially better SNR (c) than linear phase encoding. Error bars show the 95% confidence interval of the group average.

Fig. 5a and 5b show the results of ROI analysis along with two-pool model simulations of the acquisition protocol. Overall, MTR and SNR values increased with flip angle within the tested range. Centric PE images showed higher MTR values and substantially higher SNR than linear PE images (Fig. 5c). Simulated MTR values using tissue parameters from the literature agreed well with the *in vivo* values. Substitution of the observed T_2_ value of 85 ms (from fitting the T_2_ map to a single-exponential function) for the literature value (69 ms [18]) produced simulated MTR values in closer agreement with *in vivo* results.

### Accumulation of MT Effects from Prior Slices

**Fig 6:**
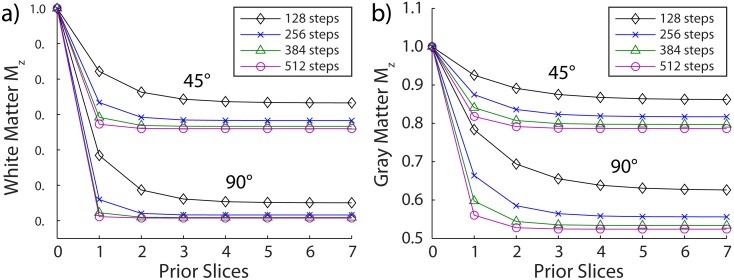
Saturation of the longitudinal magnetization accumulates over multiple prior slices with the majority of saturation due to the first prior slice. For white (a) and gray (b) matter, simulations show the longitudinal magnetization as a function of the number of prior slices for varying number of phase encoding steps per slice and for varying flip angles. One prior slice = saturation at 3840 Hz, two prior slices = saturation at 7680 Hz followed by 3840 Hz, three prior slices = saturation at 11520 Hz, 7680 Hz, and 3840 Hz, etc.

Fig. 6 shows simulations of the longitudinal magnetization (*M*_*z*, *f*_) for WM and GM for varying number of PE steps (RF-pulses) per slice, and two different flip angles. In agreement with the results in Fig. 3, the majority of saturation was generated by the first prior slice; however, a few dummy slices are needed to account for the contributions of earlier (2nd, 3rd, etc.) prior slices to reach a steady value of longitudinal magnetization across slices. More dummy slices are needed for lower flip angles and for lower number of PE steps per slice. The value of magnetization reached depended on the number of PE steps per slice and appeared to asymptotically approach true steady-state MT effects (i.e., the state that would be achieved after an infinite chain of saturation pulses at off-resonance frequency *δ*_−1_). For 128 PE steps, 5–6 dummy slices should be included to reach steady MT effects. For 256 or more PE steps, 3–4 dummy slices appeared to be sufficient. Including more dummy slices than needed would have minimal impact on scan time (∼1–3 s per dummy slice).

### Effects of Varying Interslice Delay

**Fig 7:**
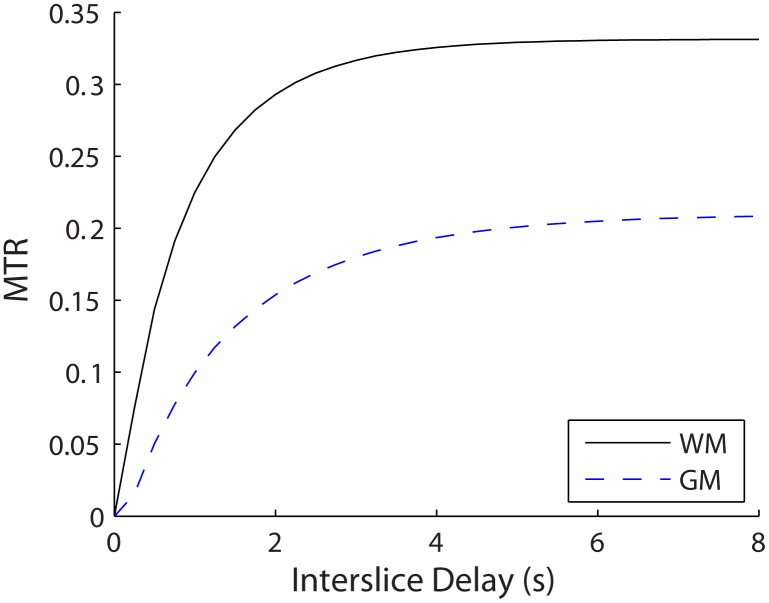
Simulated MTR values for white (solid line) and gray matter (dashed line) for varying interslice delay time for reference image acquisition. Simulations were performed using sequence parameters that matched the bSSFP acquision for images in Fig. 8.

Fig. 7 shows simulations with varying interslice delay time for acquisition of reference images. In general, the MTR asymptotically increased with interslice delay time at rate that is dependent on the T_1_ value of the tissue, with longer T_1_ values requiring a longer interslice delay to recover. An interslice delay time of 3–4 s (rather than 8 s) for acquisition of reference images would still maintain most of the MT contrast, indicating that the scan time for the acquisition of reference images could be reduced accordingly (3–5 s per slice).

### Comparison of bSSFP and SSFP-FID

**Fig 8:**
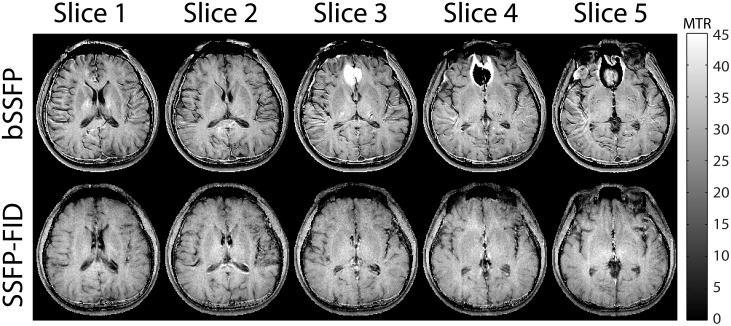
Comparison of interslice MTR imaging with bSSFP and SSFP-FID sequences. The SSFP-FID sequence significantly reduced banding artifacts in slices 3–5, but SNR was 22% lower than with bSSFP.

Fig. 8 shows representative images using the interslice MTR imaging method with bSSFP and SSFP-FID sequences. The SSFP-FID sequence is a non-balanced SSFP sequence that is less sensitive to banding artifacts in regions of high susceptibility. Regions near the sinuses in slices 3–5 were corrupted by banding artifacts with the bSSFP, but no artifacts were present in the same slices acquired with the SSFP-FID sequence. The mean MTR values of WM were 32% and 33% for bSSFP and SSFP-FID, respectively. The SNR in WM was 19.5 and 15.2 for bSSFP and SSFP-FID respectively. Overall, the SSFP-FID sequence eliminated banding artifacts, but reduced SNR by 22%.

### Interslice MTR Imaging of Meningioma

**Fig 9:**
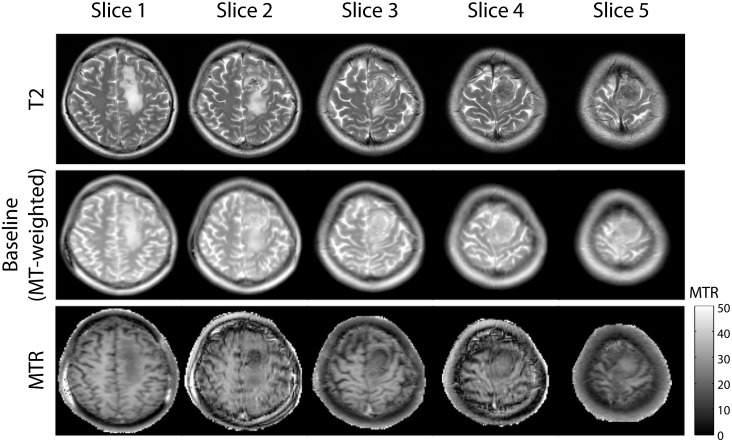
Interslice MTR images of a brain tumor (meningioma). Distinct signal characteristics in the MTR images were visible in the brain tumor regions.

Fig. 9 shows the MTR images acquired over the brain tumor region. The T_2_ images showed higher signal in the tumor region compared to normal tissue. The MTR images from the interslice method showed distinct signal characteristics in the brain tumor regions, different from the T_2_ images.

## Discussion

In this study, we demonstrated the feasibility of using interslice MT effects to generate contrast for MTR imaging. Furthermore, we validated the source of contrast as MT with phantoms and by comparing interslice MT effects with contrast generated from conventional presaturation pulses. We investigated effects of varying flip angle, number of PE steps, number of prior slices, and interslice delay. We showed that banding artifacts could be reduced by using a non-balanced SSFP sequence with no modifications to the acquisition strategy. Finally, we demonstrated the proposed method in a meningioma subject, in which the interslice MTR images showed distinct contrast.

### Interslice MTR Signal Characteristics

In the interslice method, data are acquired during the transient period of bSSFP acquisition. The magnetization state at the start of acquisition of the imaging slice (partially saturated for MT-weighted, relaxed for reference images) will move towards the steady state of the bSSFP readout, determined by the dynamics of bSSFP with contributions from on-resonance MT effects. This explains why lower MTR values were seen with linear PE. With a sufficiently large number of TRs before the acquisition of the center of k-space, no contrast is expected between the MT-weighted and reference image acquisitions. Centric PE mitigates this issue by capturing the MT contrast at the beginning of the acquisition in the low spatial frequencies. Despite this limitation, we have validated the image contrast as MT effects in phantoms (Fig. 2), shown that the contrast was predictable based on quantitative models of MT (Fig. 5), and generates distinct contrast in preliminary imaging of a meningioma subject (Fig. 9).

The results in Fig. 3 showed that the contributions from the second and third prior slices were minor compared to the first prior slice. The contributions are futher reduced in the interslice method due to nominal delay times of ∼0.65 s and ∼1.3 s for the second and third prior slices, respectively, in contrast to the first prior slice which has a nominal delay time of 0 s.

### Potential Applications

The results from imaging of the meningioma patient showed that MTR images can be acquired in a relatively short period (e.g. ∼2.1 min) over the whole brain region using the proposed interslice method. Assessment of brain tumor tissue is difficult, and the distinct MTR contrast as shown in Fig. 9 may reflect unique metabolic information of the tumor region.

The interslice MTR method may offer some advantage in terms of SAR compared to other methods, such as gradient echo methods, which rely on a separate pulse for saturation, or on-resonance MTR imaging [20, 21], which relies on a short RF-pulse duration to generate MT effects. The SAR levels for the images acquired in Fig. 8 ranged from 37–56% of the scanner limit during acquisition of MT-weighted images with a 1 ms RF-pulse; however, we have shown that high MTR values can be achieved with a longer 1.5 ms RF-pulse (Fig. 5). Since SAR levels have a quadratic dependence on RF-pulse amplitude, the interslice MTR method may be optimized for low SAR applications or high field applications by increasing the RF-pulse duration.

In the ALADDIN sequence, sequential multi-slice bSSFP acquisitions with alternating slice order and slice-select gradient polarity, as well as alternating readout gradient polarity [22] are used for interslice MT asymmetry [14] and perfusion imaging [23]. The ALADDIN sequence can be modified by adding acquisition of a set of reference images to calculate MTR images. This would allow for simultaneous acquisition of four different image contrasts: baseline bSSFP images (MT-weighted and reference), perfusion, MT asymmetry, and MTR images.

The MTR is considered a semi-quantitative measurement, since the value is dependent on scan parameters. In quantitative MT imaging, the two-pool model is fit to multiple MT-weighted acquisitions, in which parameters such as bound pool fraction (*F*) and magnetization exchange rate (*k*_*r*_) may offer better insight into tissue characteristics. Agreement of the data and two-pool model simulations in this study suggest the possibility for developing quantitative MT methods using interslice MT effects. Saturation power and off-resonance frequency can be controlled simultaneously by changing excitation pulse duration, which is inversely proportional to off-resonance frequency *δ* and inversely proportional to the square of saturation power (*W*). The technique shows potential as a novel method for fast quantitative MT, since a full set of MT-weighted images can be collected in less than 1 min.

### Comparison with On-resonsance MTR Imaging with SSFP

Another SSFP based method for MTR has previously been developed using the difference in on-resonance MT effects of long and short RF-pulse duration acquisitions [20, 21]. Thus, it is worth briefly comparing the proposed method to the on-resonance MTR method. The off-resonance signal responses of bSSFP are periodic as a function of the off-resonance frequencies and the TR of the bSSFP sequence. The on-resonance MTR method is based on the assumption that both MT-weighted (short RF-pulse) and reference (long RF-pulse) images are acquired on the pass-band region of the bSSFP off-resonance responses. This assumption seems reasonable for normal brain regions; however, the assumption may not hold under pathological conditions, such as brain tumors or hemorrhages that can cause high susceptibility effects. Because of using different TR values between MT-weighted and MT free imaging, the on-resonance MTR method also shows significantly reduced MTR in relatively short T_2_ components, as demonstrated 40% reduction of MTR in 4% agar phantom with T_1_ = 1960 ms and T_2_ = 43 ms [21]. In contrast, the interslice MTR method uses the same scan parameters for acquisition of MT-weighted and references with only the addition of an interslice delay time, which does not affect off-resonance responses of bSSFP and also provides high MTR values in relatively short T_2_ components as demonstrated in the 10% agar phantom with T_1_ = 1700 ms and T_2_ = 36 ms (Fig. 2). The interslice method may potentially offer more reliable MTR measurements in brain lesions.

In terms of sensitivity, the 3D on-resonance MTR method can provide higher SNR because of volumetric averaging effects. The proposed interslice method may be implemented in 3D as multiple overlapping thin slab acquisition, which can improve spatial resolution and SNR and requires further evaluation. Also, the availability of higher flip angle and longer data sampling time due to no restrictions on RF duration and TR in the interslice MTR method can further improves its MTR (as shown in Figs. 4 and 5) and SNR (lower acquisition BW), respectively. These factors of the interslice method can partly compensate for the volumetric averaging effects of the on-resonance MTR method.

**Fig 10:**
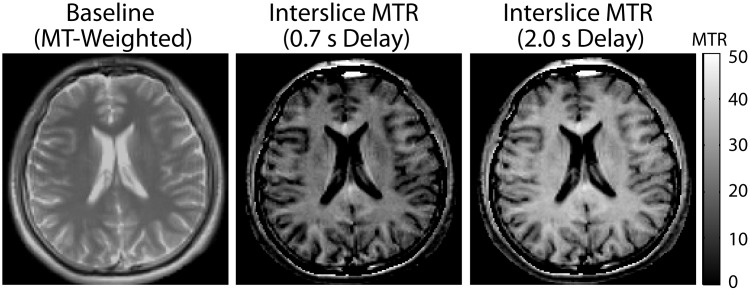
Interslice MTR images with short interslice delay times. Matrix size = 128 × 128, FA = 60°, TR/TE = 4.15/2.08 ms, RF-pulse duration = 1.24 ms, slice thickness = 5 mm, number of slices = 24 (excluding 6 dummy slices), total scan time = 56 s (0.7 s delay) and 87 (2.0 s delay). Two scans (each with 12 number of slices excluding 3 dummy slices) were spatially interleaved, in order to provide near whole brain coverage with no gap.

In terms of scan time, both the interslice and on-resonance MTR methods require covering the region of interest using bSSFP acquisitions twice (MT-weighting and MT-free). If the TR of the interslice method (e.g. 4 ms) is the same as the average of the short and long TRs of the on-resonance method (e.g. 3 ms and 5 ms [24]), the time for actual data acquisition will be similar between the two methods, except for the interslice delay required by the interslice method. Data from a single subject (Fig. 10) showed preliminary evidence that good MTR maps can be acquired with near whole brain coverage with very short interslice delay times (0.7–2.0 s), which makes the total scan time of the interslice method (1–1.5 min) about 50% longer than the on-resonance method, depending on in-plane matrix size. Note that while a short interslice delay does not provide completely MT-free reference images, the long TR (5 ms) acquisition for the on-resonance method also does not provide completely MT-free conditons. Scan time and SNR are related to each other and thus should be systematically evaluated together, but this is beyond the scope of the current study.

## Conclusions

We demonstrated the feasibility of MTR imaging using interslice MT effects generated from sequential multi-slice bSSFP acquisition. The technique provides a method for MTR imaging without additional saturation pulses. Centric PE provided higher MTR values, higher SNR, and better image contrast. Linear PE images showed image contrast influenced by relaxation effects. Simulation of the two-pool model with parameters from the literature agreed well with *in vivo* data and provided a useful tool for investigating the characteristics of interslice MT effects. The new technique could provide MTR images covering the whole brain of a tumor patient within a clinically feasible scan time of ∼2 min (or less with sequence optimization). Potential unique applications include optimization for low SAR imaging and simultaneous MTR, MT asymmetry, and perfusion imaging. Further work is needed to systematically compare the proposed method with on-resonance MTR method and evaluate clinical usefulness of the proposed method.
